# Interim Positron Emission Tomography–Guided Dose-Adapted Residual Site Radiation Therapy Improves Survival in Diffuse Large B-Cell Lymphoma Patients With Partial Metabolic Response After R-CHOP: A Retrospective Cohort Analysis

**DOI:** 10.1016/j.adro.2026.102078

**Published:** 2026-05-23

**Authors:** Jie Zhao, Fang Huang, Rui Liu, Jiangbo Wan, Wenhao Zhang, Zhichao Li

**Affiliations:** a Department of Hematology, Xinhua Hospital Affiliated to Shanghai Jiaotong University School of Medicine, Shanghai, China; b Department of Oncology, Fudan University Shanghai Cancer Center, Shanghai, China

## Abstract

**Purpose:**

Optimal management of diffuse large B-cell lymphoma (DLBCL) patients achieving partial remission (Deauville 5-point scale [5PS]: 4-5) with interim positron emission tomography (iPET) is undefined. This study evaluated iPET-guided, dose-adapted residual site radiation therapy (RSRT) outcomes.

**Methods and Materials:**

Retrospective analysis of 68 DLBCL patients with iPET 5-PS 4 to 5 after 4 Rituximab, Cyclophosphamide, Doxorubicin, Vincristine, and Prednisone (R-CHOP) cycles was conducted. Twenty-nine patients received 2 additional R-CHOP + risk-adapted RSRT (24 Gy for 5-PS4; 40 Gy for 5-PS5), while 39 received 2 to 4 R-CHOP cycles without radiation therapy (RT).

**Results:**

The RT cohort demonstrated significantly higher end-of-treatment complete response rates (72.4% vs 38.5%; *P* = .006) and superior progression-free survival (PFS) (2-year, 85.8% vs 52.9%; 5-year, 57.2% vs 44.1%; log-rank *P* = .025). No overall survival difference was observed (0 deaths in RT vs non-RT cohort; 2- and 5-year overall survival rates of 88.1% and 77.3%, respectively; *P* = .068). Multivariate analysis confirmed RT as an independent protective factor (hazard ratio, 0.26; 95% CI, 0.09-0.79; *P* = .018). Subgroup analysis revealed consistent PFS benefits across high-risk features, including advanced stage, high International Prognostic Index, and *BCL2+* disease. RT-related toxicities were manageable (grade 4 gastrointestinal perforation, n = 1; grade 2 pneumonitis, n = 2).

**Conclusions:**

iPET-guided dose-adapted RSRT significantly enhances tumor response and PFS in DLBCL patients with suboptimal interim metabolic response, demonstrating favorable safety. This represents a promising strategy for high-risk partial metabolic responders, warranting prospective validation.

## Introduction

Diffuse large B-cell lymphoma (DLBCL) represents the most prevalent subtype of non-Hodgkin lymphoma among the adult population.^1^ The R-CHOP immunochemotherapy regimen, comprising rituximab, cyclophosphamide, doxorubicin, vincristine, and prednisone, constitutes the established first-line therapeutic standard for newly diagnosed DLBCL patients. While over half of patients achieve clinical remission following R-CHOP therapy, a substantial proportion (30%-40%) experience unfavorable outcomes, primarily manifesting as primary refractory disease or early relapse postinitial treatment.^2^^,^^3^ Interim ^18^F-fluoro-2-dexoxy-D-glucose–positron emission tomography (iPET) has emerged as a critical modality for dynamic therapeutic response assessment. Its utility as an independent prognostic indicator, particularly for identifying high-risk patient subsets, has been robustly demonstrated. Patients attaining complete remission (CR) on iPET have a significantly superior prognoses.^4^, ^5^, ^6^ Conversely, iPET revealing progressive disease (PD) necessitates treatment intensification. However, for patients demonstrating partial remission (PR) on iPET, optimal subsequent management strategies remain heterogeneous due to a lack of definitive consensus guidelines. Patients with persistently positive lesions detected on iPET exhibit a substantially elevated risk of disease progression and relapse.^7^^,^^8^

Given that residual disease in this subset is frequently localized, enhanced local control with radiation therapy (RT) has the potential to translate into improved overall outcomes. RT constitutes a significant therapeutic modality in the management of DLBCL. Evidence suggests that consolidative RT following achievement of CR with R-CHOP immunochemotherapy in patients presenting with bulky disease or advanced-stage DLBCL may confer an overall survival (OS) benefit of up to 15%, accompanied by significant improvements in progression-free survival (PFS).^9^^,^^10^ A large-scale cohort study by Freeman et al^11^ of 723 patients with advanced-stage DLBCL demonstrated that nonprogressing patients with anatomically suitable disease who received 6 to 8 cycles of R-CHOP and exhibited a positive end-of-therapy (EOT) positron emission tomography (PET) scan yet underwent selective RT attained outcomes comparable to those achieving a negative EOT-PET.

However, prospective data specifically evaluating the benefit of consolidative RT in patients achieving only PR on interim PETcomputed tomography (CT) remain scarce. Furthermore, the risk of treatment-related toxicities associated with RT necessitates careful consideration in clinical decision-making, highlighting the critical need to optimize therapeutic efficacy while minimizing radiation-induced adverse effects. The integration of PET-CT into treatment planning has significantly enhanced the precision of radiation target volume delineation in lymphoma. By enabling accurate identification of individual sites of disease involvement, PET-CT facilitates a substantial reduction in the radiation field size. In the context of modern response-adapted strategies, consolidative RT delivered to these smaller, PET-defined volumes after systemic therapy can be prescribed at reduced doses, typically 30 Gy, without compromising disease control while markedly lowering the risk of acute and late toxicities.^12^ To optimize therapeutic efficacy and minimize treatment-related morbidity, our study employed PET-directed residual site RT (pRSRT), consistent with contemporary guidelines and the standardized National Clinical Trials Network radiation target volume nomenclature.^13^

Using PET-based response criteria (specifically the Deauville 5-point scale [5PS]), this study pioneers a stratified RT regimen: administering 24 Gy for to patients scoring 5PS of 4 and escalating to 40 Gy for those scoring 5PS of 5. This retrospective evaluation aimed to elucidate the survival benefit of pRSRT in DLBCL patients exhibiting iPET partial responders (iPET-PR), thereby generating crucial evidence to inform the optimization of individualized treatment strategies.

## Methods and Materials

### Patient selection

Clinical data were retrospectively collected from DLBCL patients treated between August 2019 and October 2024, excluding those with primary central nervous system or primary mediastinal DLBCL or with a compromised baseline status precluding standard immunochemotherapy. All enrolled patients received first-line R-CHOP therapy. iPET was performed after 4 treatment cycles and was categorized per Cheson et al^14^ criteria as CR, PR, stable disease, or PD. Sixty-eight patients with PR comprised the study cohort; 29 subsequently received 2 additional R-CHOP cycles plus pRSRT (RT cohort), whereas 39 received 2 to 4 additional R-CHOP cycles without RT (non-RT cohort). RT cohort received pRSRT in accordance with the National Clinical Trials Network radiation target volume nomenclature.^13^ pRSRT uses interim or EOT-PET-CT imaging to guide the selection of residual disease sites at high risk of relapse for targeted RT. In this study, radiation target volumes were delineated based on findings from iPET scans, with the intent to deliver consolidative RT to metabolically active residual lesions at the highest risk of recurrence. Within the RT cohort, RT dose was stratified by 5PS scores: patients scoring 4 received 24 Gy, whereas those scoring 5 received 40 Gy. Comprehensive clinical, pathologic, and radiological data were systematically documented for all subjects.

### Endpoints and definitions

Patient follow-up was conducted via telephonic interviews and medical record review until the landmark date of June 30, 2025. PFS was defined as the time from treatment initiation to the first occurrence of disease progression, relapse, or death from any cause. OS was defined as the duration from treatment commencement to death from any cause or to the last follow-up date, whichever occurred first. This study received approval from the Institutional Review Board of Xinhua Hospital, Shanghai Jiao Tong University School of Medicine (approval number: XHEC-D-2025-120), and was conducted in accordance with institutional review board standards for human subjects research and the ethical principles of the Declaration of Helsinki. Informed consent was waived because of the retrospective design of the study.

### Statistical analysis

Statistical analyses were performed using SPSS software (version 26.0; IBM Corporation) and the R statistical computing environment (version 4.4.2; R Foundation for Statistical Computing). Categorical variables were presented as frequencies with corresponding percentages and compared using either Pearson’s χ^2^ test or Fisher’s exact test, as appropriate. Survival outcomes were analyzed using the Kaplan-Meier method, with between-group comparisons assessed by the log-rank test; prognostic factors were evaluated using both univariate and multivariate Cox proportional hazards regression models. Forest plots were generated to visualize findings from subgroup analyses, with all statistical significance determined at a 2-sided alpha level of *P* < .05.

## Results

### Patient characteristics

The clinical characteristics of the 68 enrolled DLBCL patients are detailed in Table 1, demonstrating comparable baseline distributions between the RT and non-RT cohorts. The RT cohort (n = 29) exhibited a predominance of patients aged >60 years, men, with a non-GCB histologic subtype, and B symptoms (fever, night sweats, and weight loss) at initial diagnosis. Advanced Ann Arbor stage (III-IV) was observed in 25 patients (86.2%), an 75.9% elevated International Prognostic Index (IPI) score (3-5) and 72.4% having elevated lactate dehydrogenase (LDH), with bulky (≥10 cm) disease present in 5 cases (17.2%). Immunohistochemical analysis revealed *MYC+* expression in 16 patients (55.2%), *BCL2+* in 24 (82.8%), and *BCL6+* in 21 (72.4%), while Ki-67 ≥ 80% was detected in 15 patients (51.7%). iPET assessment used the 5PS yielded scores of 4 in 17 RT patients (58.6%), compared with 21 patients (53.8%) in the non-RT cohort.

**Table 1:** Clinical characteristics and outcomes of diffuse large B-cell lymphoma patients receiving radiation therapy versus nonradiation therapy patients

Variables	Number of patients, n (%)		*P* value
	Radiation therapy cohort (n = 29)	Nonradiation therapy cohort (n = 39)	
Sex			.919
Male	16 (55.2)	22 (56.4)	
Female	13 (44.8)	17 (43.6)	
Age (y)			.119
≤60	10 (34.5)	7 (17.9)	
>60	19 (65.5)	32 (82.1)	
Hans classification			.418
GCB	14 (48.3)	15 (38.5)	
Non-GCB	15 (51.7)	24 (61.5)	
MYC			.457
*MYC+*	16 (55.2)	25 (64.1)	
*MYC-*	13 (44.8)	14 (35.9)	
BCL2			.611
*BCL2+*	24 (82.8)	34 (87.2)	
*BCL2-*	5 (17.2)	5 (12.8)	
BCL6			.612
*BCL6+*	21 (72.4)	26 (66.7)	
*BCL6-*	8 (27.6)	13 (33.3)	
Ki67			.971
Ki67 ≥ 80%	15 (51.7)	20 (51.3)	
Ki67 < 80%	14 (48.3)	19 (48.7)	
B symptoms			.054
No	11 (37.9)	24 (61.5)	
Yes	18 (62.1)	15 (38.5)	
Ann Arbor stage			.645
Ⅰ-Ⅱ	4 (13.8)	7 (17.9)	
Ⅲ-Ⅳ	25 (86.2)	32 (82.1)	
IPI score			.721
0-2	7 (24.1)	8 (20.5)	
3-5	22 (75.9)	31 (79.5)	
LDH, U/L			.176
≤211	8 (27.6)	17 (43.6)	
>211	21 (72.4)	22 (56.4)	
5PS			
4	17 (58.6)	21 (53.8)	.695
5	12 (41.4)	18 (46.2)	
Bulk, cm			.733
≤10	24 (82.8)	34 (87.2)	
>10	5 (17.2)	5 (12.8)	

*Abbreviations:* 5PS = 5-point Deauville scale; IPI = International Prognostic Index; LDH = lactate dehydrogenase; GCB = germinal center B-cell-like.

### Survival analyses

In the EOT assessment, the RT cohort demonstrated significantly higher objective response rates compared with the non-RT cohort, with complete response rates of 72.4% vs 38.5% (*P* = .006), and overall response rates of 86.2% vs 56.4% (*P* = .009). In the EOT-PET assessment, response distributions differed significantly between cohorts. In the non-RT cohort (n = 39), 16 patients (41.0%) achieved CR, 10 (25.6%) had PR, and 13 (33.3%) showed PD. In contrast, among the 29 patients receiving pRSRT, 21 (72.4%) attained CR, 4 (13.8%) had PR, and 4 (13.8%) progressed. Pearson’s χ^2^ test revealed that RT was significantly associated with higher EOT CR rates (72.4% vs 41.0%; *P* = .014). The benefit of RT was most evident in the high-risk subgroup with interim 5PS score of 5: EOT CR rates were 83.3% (10/12) in the RT group versus 22.2% (4/18) in the non-RT group (*P* = .002) (Table 2).

**Table 2:** Comparison of end-of-treatment positron emission tomography complete response rates between radiation therapy and nonradiation therapy cohorts

Subgroup	Cohort	CR cases/total (n)	CR rate (%)	*P* value
Overall cohort	Nonradiation therapy cohort	16/39	41	.014
	Radiation therapy cohort	21/29	72.4	-
5PS 5	Nonradiation therapy cohort	4/18	22.2	.002
	Radiation therapy cohort	10/12	83.3	-
5PS 4	Nonradiation therapy cohort	12/21	57.1	.744
	Radiation therapy cohort	11/17	64.7	-

*Abbreviations:* 5PS = Deauville 5-point scale; CR = complete response.

**Figure 1:**
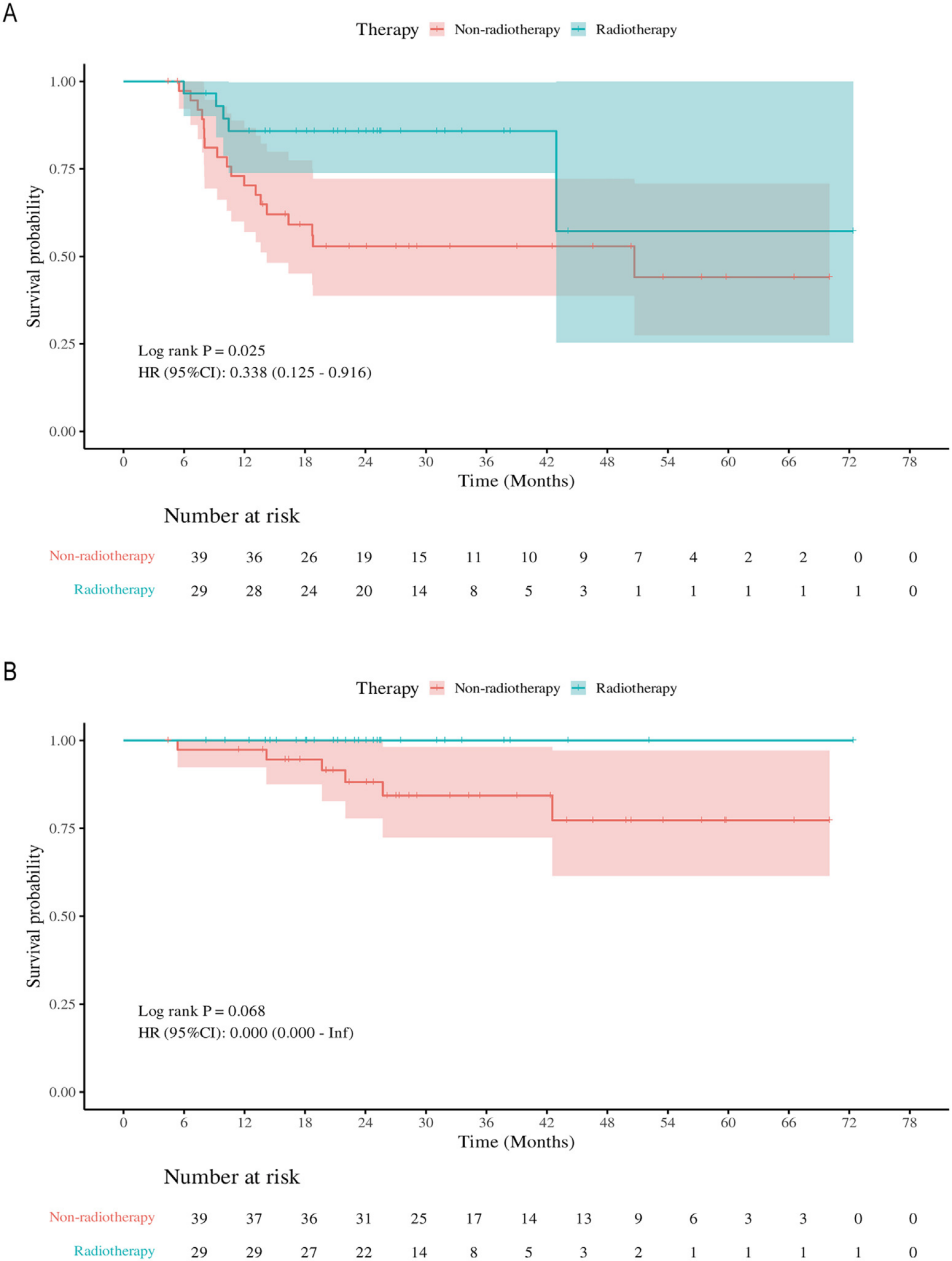
Survival outcomes of diffuse large B-cell lymphoma patients with interim positron emission tomography Deauville 5-point scale scores of 4 to 5 partial remission. (A) Progression-free survival (PFS) in radiation therapy versus nonradiation therapy cohorts. The radiation therapy cohort showed significantly superior 2-year (85.8% vs 52.9%) and 5-year PFS (57.2% vs 44.1%; log-rank *P* = .025). (B) Overall survival (OS) in radiation therapy versus nonradiation therapy cohorts. No statistically significant difference was observed (2-year OS, 100% vs 88.1%; 5-year OS, 100% vs 77.3%; log-rank P = .068). *Abbreviations:* HR = hazard ratio.

Survival analyses revealed superior PFS in the RT group (2-year PFS, 85.8%; 5-year PFS, 57.2%) compared with the non-RT group (2-year PFS, 52.9%; 5-year PFS, 44.1%; log-rank *P* = .025; Fig. 1A). While no mortality events occurred in the RT cohort during the follow-up period, the non-RT group exhibited 2- and 5-year OS rates of 88.1% and 77.3%, respectively (log-rank *P* = .068; Fig. 1B). Notably, the Kaplan-Meier PFS curves demonstrated an early divergence between the RT and non-RT groups. Multivariate Cox regression identified RT as the sole independent protective factor for PFS (hazard ratio, 0.26; 95% CI, 0.09-0.79; *P* = .018) (Table 3). Conversely, multivariate OS analysis yielded no statistically significant associations, attributable to limited mortality events (particularly the absence of deaths in the RT cohort) during the observation window (Table E1).

**Table 3:** Univariable and multivariate Cox regression analysis for progression-free survival

	Univariable		Multivariate	
Variables	HR (95% CI)	*P* value	HR (95% CI)	*P* value
Radiation therapy	0.34 (0.12-0.92)	.033	0.26 (0.09-0.79)	.018
Male	0.72 (0.32-1.64)	.441	0.66 (0.24-1.83)	.421
≤60 y	1.21 (0.48-3.07)	.688	2.75 (0.81-9.36)	.105
Non-GCB	2.27 (0.89-5.75)	.085	2.73 (0.83-8.95)	.097
*MYC+*	0.89 (0.39-2.03)	.775	0.88 (0.27-2.82)	.825
*BCL2+*	1.38 (0.41-4.73)	.603	0.46 (0.08-2.67)	.383
*BCL6+*	1.54 (0.60-3.90)	.367	2.01 (0.68-5.89)	.205
Ki67 ≥80%	1.13 (0.50-2.57)	.766	1.05 (0.38-2.93)	.926
B symptoms	1.23 (0.54-2.78)	.626	1.27 (0.46-3.48)	.644
Ann Arbor stage Ⅲ-Ⅳ	1.68 (0.50-5.67)	.406	0.81 (0.16-4.21)	.803
IPI 3-5 scores	2.25 (0.66-7.65)	.193	4.62 (0.56-37.77)	.154
LDH ≤ 211 U/L	1.27 (0.55-2.95)	.576	2.49 (0.82-7.53)	.106
Bulk (>10 cm)	1.10 (0.32-3.74)	.878	1.16 (0.25-5.33)	.851
5PS 5	2.51 (1.06-5.92)	.036	1.68 (0.52-5.50)	.389

*Abbreviations:* 5PS = Deauville 5-point scale; IPI = International Prognostic Index; LDH = lactate dehydrogenase; GCB = germinal center B-cell-like.

Given the clinical diversity of our cohort, we performed exploratory subgroup analyses by Ann Arbor stage and tumor bulk. RT was associated with significantly improved PFS in patients with Ann Arbor stage III to IV disease (log-rank *P* = .017; Fig. E1). Notably, while no significant survival benefit was observed in patients with bulky disease, those without bulky disease showed a marked PFS advantage with RT (*P* = .007; Fig. E2).

### RT safety profile and subgroup forest plot analysis

In the RT cohort, 1 patient who received 40 Gy developed grade 3 myelosuppression, while 1 patient who received 24 Gy exhibited grade IV myelosuppression; the remaining patients demonstrated grade ≤2 myelosuppression. Regarding nonhematological toxicities, grade 4 radiation-induced bowel injury (intestinal perforation) and grade 2 radiation pneumonitis occurred in 1 patient each in the 40 Gy group, whereas the 24 Gy group had 1 case of grade 2 radiation pneumonitis; no other severe (grade ≥3) radiation-related toxicities were observed in the cohort, indicating an overall manageable RT safety profile.

**Figure 2:**
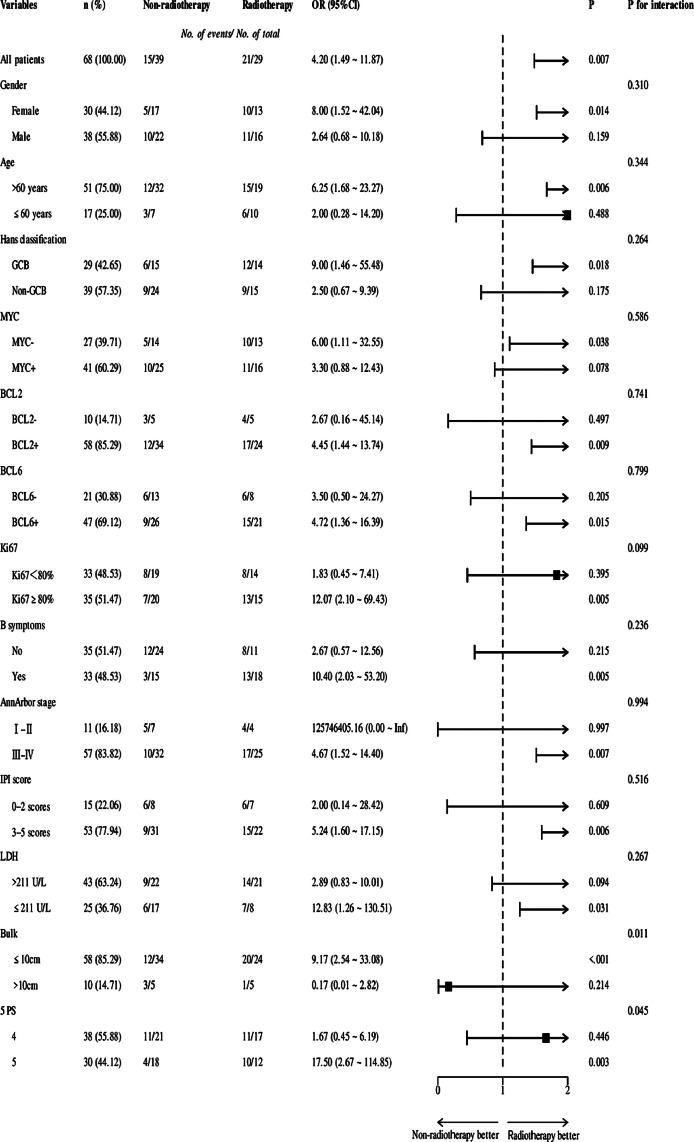
Forest plots of complete response rate subgroup analyses in diffuse large B-cell lymphoma patients. Reference line: odds ratio (OR) = 1 (no difference); horizontal line = 95% CI. Subgroup definitions: female, age >60 years, B symptoms, advanced stage (Ann Arbor III-IV), high International Prognostic Index (IPI; score 3-5), *BCL2+*, and Ki-67 ≥ 80%. All subgroups consistently favored radiation therapy. *Abbreviations*: 5PS = Deauville 5-point scale; LDH = lactate dehydrogenase; GCB = germinal center B-cell-like.

**Figure 3:**
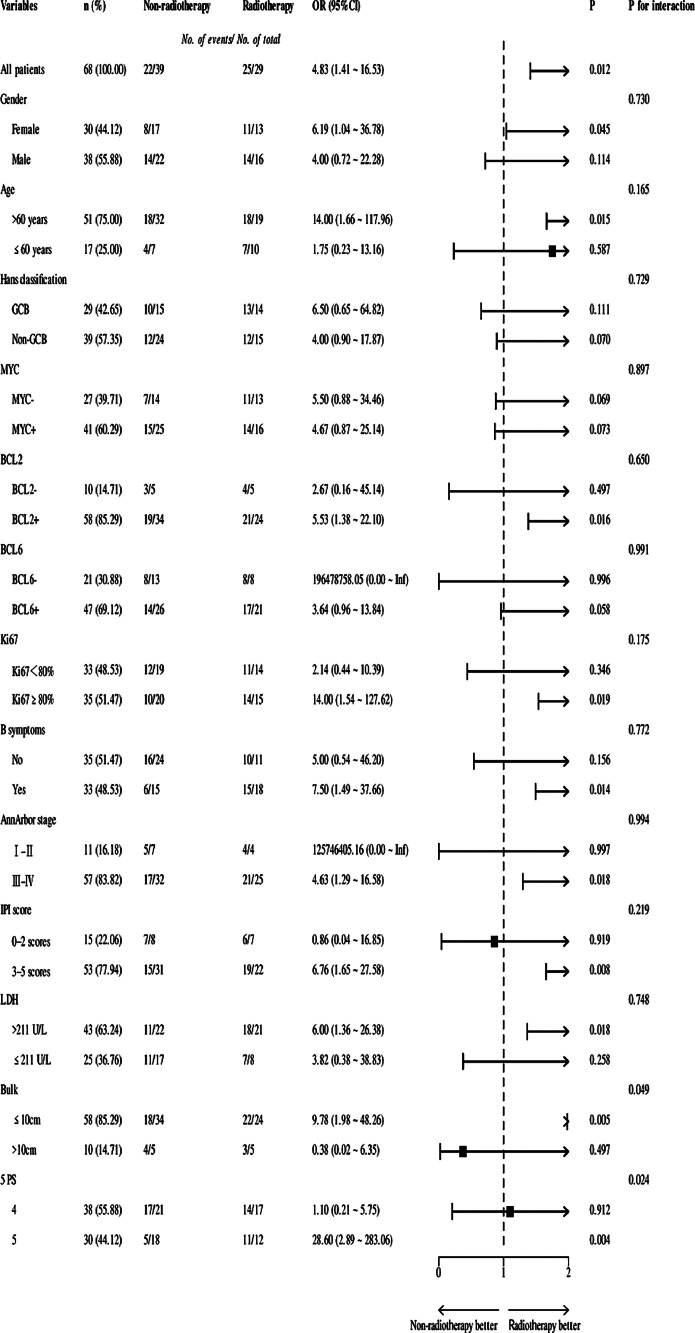
Forest plots of overall response rate subgroup analyses in diffuse large B-cell lymphoma patients. Reference line: odds ratio (OR) = 1 (no difference); horizontal line = 95% CI. Subgroup definitions: female, age >60 years, B symptoms, advanced stage (Ann Arbor III-IV), high International Prognostic Index (IPI score, 3-5), *BCL2+*, Ki-67 ≥ 80%. All subgroups consistently favored radiation therapy. *Abbreviations*: 5PS = Deauville 5-point scale; LDH = lactate dehydrogenase; GCB = germinal center B-cell-like.

Subsequent forest plot analysis stratified by treatment cohorts (Figure 2, Figure 3) demonstrated that significant improvements in complete response rate were consistently observed in female patients, aged >60 years, those with the GCB subtype, expressing *MYC-, BCL2+*, and *BCL6+*, with Ki-67 ≥ 80%, B symptoms, advanced Ann Arbor stage (III-IV), an IPI score of 3 to 5, and with normal LDH levels. Furthermore, RT significantly enhanced overall response rates in female patients, individuals aged >60 years, those expressing *BCL2+*, with Ki-67 ≥ 80%, B symptoms, with Ann Arbor stage III to IV, an IPI score of 3 to 5, and with elevated LDH levels (all between-group *P* values < .05).

## Discussion

Despite therapeutic advances in the R-CHOP era, approximately 20% to 40% of DLBCL patients experience treatment failure requiring salvage therapy,^15^ with a subset exhibiting rapid relapse within 2 years of initial remission; consequently, early identification of suboptimal responders necessitating treatment intensification remains a pivotal clinical challenge. PET/CT quantitatively assesses metabolic activity using the Deauville scoring system, serving as a critical noninvasive modality for visualizing treatment response throughout lymphoma management. Patients achieving EOT-PET–negative status demonstrate significantly prolonged PFS and overall OS, whereas EOT-PET–positive status warrants therapeutic escalation; however, the prognostic utility of iPET in DLBCL remains incompletely defined,^16^^,^^17^ and optimal management strategies for iPET-PR lack consensus—implementing Deauville score-directed therapeutic interventions for iPET-PR patients to facilitate early conversion to EOT-PET–negative status may represent a critical opportunity for prognostic enhancement in this subpopulation.

A 5PS score of 5 strongly indicates metabolically active residual disease, warranting aggressive intervention, including systemic therapy with or without consolidative RT. In contrast, DS4 lesions may represent residual inflammation, immune cell infiltration, or low-grade metabolic activity rather than viable lymphoma.^18^ Moskowitz et al^19^ demonstrated that many iPET-positive lesions (DS4-5) are histologically negative, highlighting diagnostic ambiguity. Although serial iPET reassessment could theoretically clarify lesion biology, it introduces clinically meaningful delays (4-6 weeks) during which true PD may advance unchecked—particularly concerning in aggressive lymphomas. Moreover, biopsy of residual sites is often impractical due to anatomic inaccessibility, sampling error, procedural invasiveness, and potential treatment delays, driving a preference for noninvasive imaging assessment.

RT demonstrates established efficacy in relapsed/refractory DLBCL, improving local control when added to second-line therapy.^20^, ^21^, ^22^ Freeman et al^11^ reported that consolidative RT for nonprogressing EOT-PET–positive patients after ≥6 R-CHOP cycles yielded outcomes comparable to EOT-PET–negative patients (3-year OS ≈80%). For patients with localized ^18^F-fluoro-2-dexoxy-D-glucose–avid disease after initial chemotherapy, RT may provide curative potential; however, RT carries risks of cardiac mortality, myelosuppression, and secondary malignancies,^23^ with toxicity profiles dictated by irradiated anatomy and dose/fractionation. This concern motivates dose de-escalation efforts to mitigate toxicity.^24^ Modern RT has evolved toward precision and toxicity mitigation. A critical methodological consideration in our study pertains to the timing of PET-CT guidance in RT decision-making. We acknowledge the inherent limitations of iPET, particularly its reduced specificity for distinguishing residual lymphoma from posttreatment inflammation compared with EOT-PET-CT. Accordingly, proponents of EOT-PET–guided approaches argue that deferring radiation decisions until completion of systemic therapy may optimize patient selection, as exemplified by the EOT-PET–guided strategy reported by Freeman et al.^11^ Nevertheless, emerging evidence supports the feasibility of iPET-guided adaptive strategies, which offer the potential advantage of earlier treatment modification while significantly reducing treatment-related toxicity.^25^

Our risk-adapted approach—24 Gy for DS4 versus 40 Gy for DS5—is grounded in both biological rationale and clinical pragmatism. Freeman et al's^11^ EOT-PET–positive cohort received 30 to 40 Gy,^26^^,^^27^ and the randomized trial of nonbulky, limited-stage DLBCL demonstrated a 92% (95% CI, 89.6%-94.4%) 5-year event-free survival with R-CHOP plus 40 Gy involved-field RT.^28^ Furthermore, accumulating data indicate that, in the context of effective systemic therapy and refined chemotherapy response assessment via PET-CT, RT doses in combined-modality treatment programs may be safely de-escalated to 20 Gy, achieving comparable local control with diminished acute and long-term adverse effects.^29^ Aligning with International Lymphoma Radiation Oncology Group guidelines^20^ and prior evidence,^12^^,^^29^^,^^30^ we administered 40 Gy for a 5PS score of 5, reflecting high residual activity, whereas 24 Gy was administered for a 5PS score of 4, reflecting a lower metabolic burden. It is important to clarify that the use of 24 Gy in our study is exploratory, which requires validation in prospective trials.

Our findings confirm significant clinical benefits of risk-adapted RT stratification, demonstrating that 72.4% of iPET-PR patients achieved EOT-PET negativity following consolidation RT, compared with 41% in nonirradiated controls (*P* = .014). The benefit of RT was most evident in the high-risk subgroup with interim 5PS score of 5: EOT CR rates were 83.3% (10/12) in the RT group versus 22.2% (4/18) in the non-RT group (*P* = .002). RT-treated patients exhibited superior PFS, with 2- and 5-year rates of 85.8% versus 52.9%, and 57.2% versus 44.1%, respectively (*P* = .025), with multivariate analysis identifying , RT as the sole independent protective factor for PFS (hazard ratio, 0.26; 95% CI, 0.09-0.79; *P* = .018). Notably, the Kaplan-Meier PFS curves demonstrated an early divergence between the RT and non-RT groups, suggesting that pRSRT may help sustain CR and reduce the risk of early disease progression. This pattern supports the potential role of RT as a local consolidative strategy to deepen and prolong the response in selected patients. Mechanistically, this survival benefit appears to correlate with enhanced metabolic clearance observed on interim imaging, supporting the hypothesis that RT facilitates a crucial conversion from partial to complete response. Notably, no deaths occurred in the RT cohort through follow-up termination, and toxicity profiles remained manageable with only 1 grade 4 radiation-induced perforation requiring surgical intervention and 2 grade 2 pneumonitis cases (not requiring respiratory support); no other severe radiation-related toxicities were observed, confirming an acceptable safety profile.

Notably, patients without bulky disease showed a marked PFS advantage with RT, whereas no significant survival benefit was observed in those with bulky disease in our study. This outcome may reflect selective RT use in nonbulky patients with persistent PET positivity or suboptimal response, where localized consolidation effectively eradicated residual disease with smaller, more tolerable target volumes. Although the lack of benefit in bulky disease aligns with Freeman et al’s^11^ findings that PET-negative patients have excellent prognosis regardless of tumor bulk, it likely reflects limited statistical power because of small subgroup size rather than absence of true effect. Although exploratory subgroup analyses suggested potential associations between RT benefit and factors including age, disease stage, IPI score, and cell-of-origin subtype, these observations remain hypothesis-generating given the study’s retrospective nature and limited sample size.

While our findings support the role of pRSRT in the R-CHOP era for patients with PR, we recognize the transformative impact of contemporary regimens, including frontline Pola-R-CHP (Polatuzumab vedotin, Rituximab, Cyclophosphamide, Doxorubicin, and Prednisone) and novel salvage therapies such as bispecific antibodies, antibody-drug conjugates, and CAR T (Chimeric Antigen Receptor T-cell) Immunotherapy. Further research is needed to define the evolving role of RT in these modern treatment paradigms, particularly for patients who are incomplete metabolic responders. Concurrently, emerging advances in DLBCL risk stratification—as highlighted in the 2024 American Journal of Hematology update,^31^—propose artificial intelligence–driven quantification of PET/CT parameters, such as total metabolic tumor volume, to enhance risk assessment and disease surveillance. These novel metabolic biomarkers hold promise for refining patient selection for RT intensification.

While the dose and volume used in this study are not yet considered standard for frontline consolidative RT, our findings highlight significant institutional practice variations and suggest favorable outcomes with this de-escalation approach. However, it is important to acknowledge that our study is limited by a modest cohort size, with approximately 30 to 40 patients in each treatment group. This restricts the statistical power of subgroup analyses; consequently, all subgroup findings—including those regarding bulky versus nonbulky disease—must be interpreted as hypothesis-generating and exploratory rather than definitive. Despite these limitations, the observed patterns support the potential role of tailored RT strategies. Larger prospective studies are urgently needed to validate these observations and to establish whether such de-escalated protocols can be safely adopted as a new standard of care.

## Conclusions

Collectively, this study substantiates the clinical feasibility of selective residual-site RT guided by iPET assessment in DLBCL patients achieving partial metabolic response after R-CHOP, demonstrating association with favorable survival outcomes. Furthermore, the implemented response-adapted RT dosing paradigm exhibited a manageable safety profile, with severe radiation toxicities occurring infrequently and most patients tolerating treatment.

## Disclosures

The authors declare that they have no known competing financial interests or personal relationships that could have appeared to influence the work reported in this paper.

## Appendix Supplementary materials

Supplementary table1 Image, application 1

Figure S1 Image, image 2 dummy alt text

Figure S2 Image, image 3 dummy alt text

## Footnotes

Supplementary material associated with this article can be found in the online version at doi:10.1016/j.adro.2026.102078.
