# Supplementary figures and images for: Interim Positron Emission Tomography–Guided Dose-Adapted Residual Site Radiation Therapy Improves Survival in Diffuse Large B-Cell Lymphoma Patients With Partial Metabolic Response After R-CHOP: A Retrospective Cohort Analysis
Source: Adv Radiat Oncol. 2026 May 23;11(10):102078. doi: 10.1016/j.adro.2026.102078 (PMC13333299; doi:10.1016/j.adro.2026.102078)

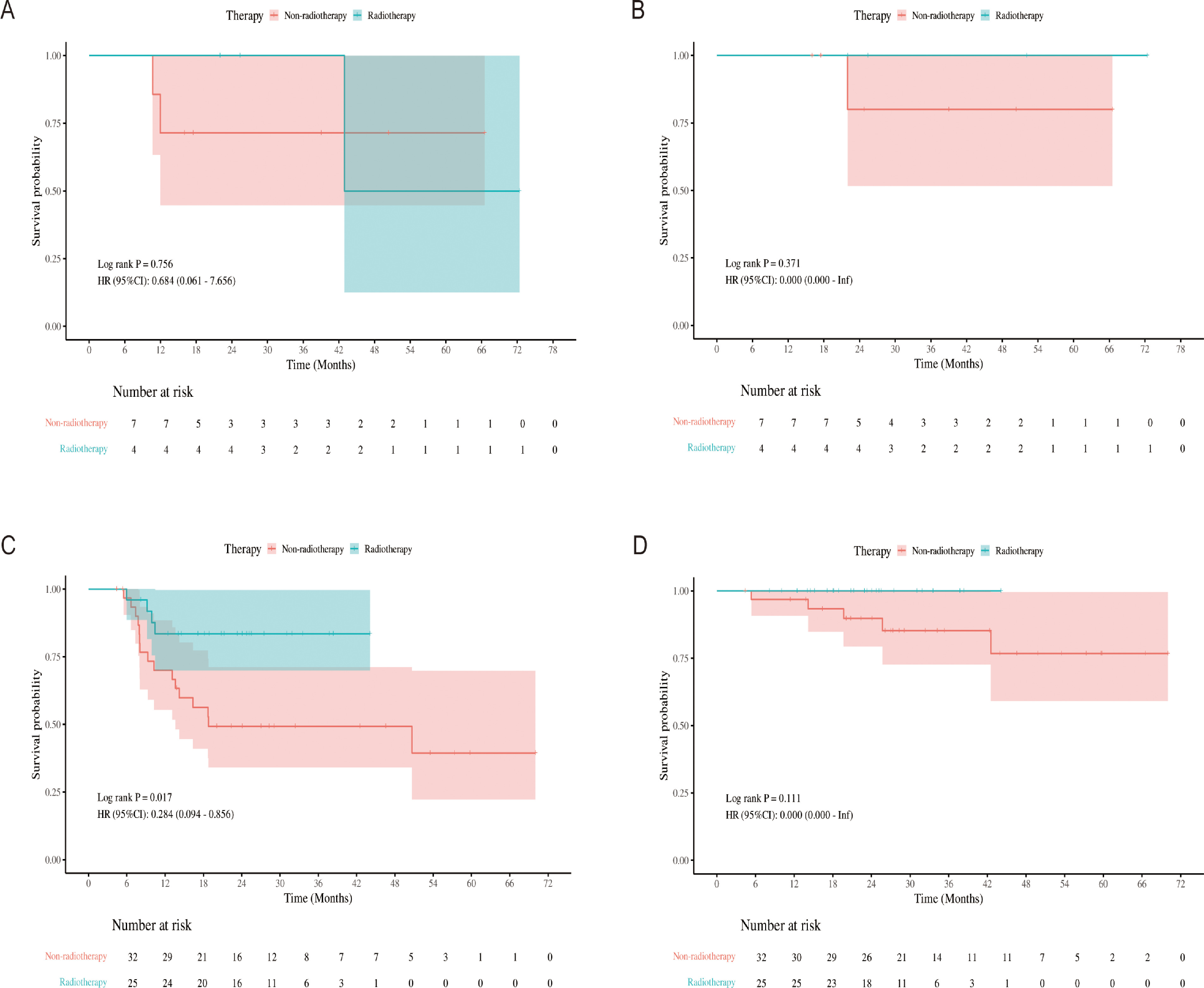

Supplement: Figure S1 [file mmc2.jpg]

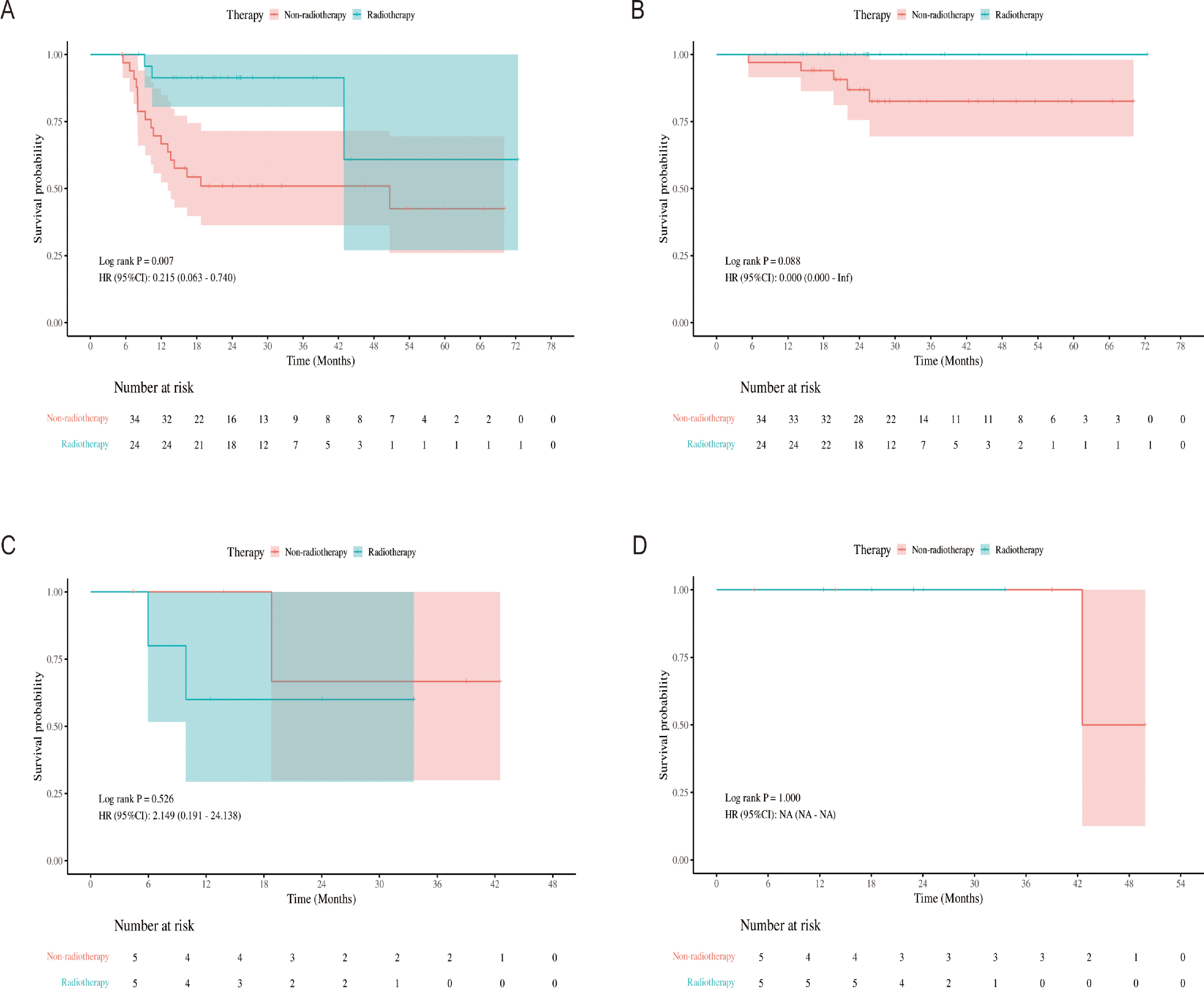

Supplement: Figure S2 [file mmc3.jpg]
